# Supplementary material for: Bacterioneuston in Lake Baikal: Abundance, Spatial and Temporal Distribution
Source: Int J Environ Res Public Health. 2018 Nov 19;15(11):2587. doi: 10.3390/ijerph15112587 (PMC6266163; doi:10.3390/ijerph15112587)
Supplement: Supplementary file 1 [file ijerph-15-02587-s001.pdf]

International Journal of
*Environmental Research and Public Health*

MDPI

**Table S1.** Location of the SML and UW sampling stations in Lake Baikal in 2013–2016.

| No. | Sampling Station | Latitude (N) | Longitude (E) |
|---|---|---|---|
| 1 | 12 km from Kultuk village | 51°40.631′ | 103°52.461′ |
| 2 | 3 km from Solzan village | 51°31.818′ | 104°13.808′ |
| 3 | central station of section Marituy village–Solzan village | 51°38.808′ | 104°13.642′ |
| 4 | 3 km from Maritui village | 51°45.575′ | 104°13.170′ |
| 5 | central station of section Ivanovskii Cape−Murino village | 51°38.761′ | 104°25.827′ |
| 6 | Listvenichnyi Bay | 51°50.648′ | 104°49.652′ |
| 7 | central station of section Listvyanka village–Tankhoi village | 51°42.330′ | 105°00.597′ |
| 8 | 3 km from Tankhoi village | 51°35.510′ | 105°06.747′ |
| 9 | opposite Bolshie Koty village | 51°53.432′ | 104°58.841′ |
| 10 | opposite Babyshkin village | 51°45.012′ | 105°50.141′ |
| 11 | Peschanaya Bay | 52°11.708′ | 105°40.880′ |
| 12 | Babushka Bay | 52°15.592′ | 105°42.453′ |
| 13 | 1 km from Kharauz branch (Selenga River) | 52°17.609′ | 106°14.732′ |
| 14 | 3 km from Kharauz branch (Selenga River) | 52°17.485′ | 106°12.629′ |
| 15 | central station of section the Anga River−Sukhaya River | 52°39.914′ | 106°51.321′ |
| 16 | Mukhor Bay (Maloe More strait) | 53°02.102′ | 106°46.428′ |
| 17 | Bazarnaya Bay (Olkhonskie Vorota strait) | 53°01.356′ | 106°52.193′ |
| 18 | central station of Olkhonskie Vorota strait | 53°01.566′ | 106°54.293′ |
| 19 | central station of Maloe More strait | 53°14.546′ | 107°15.242′ |
| 20 | opposite Zunduk Cape (Maloe More strait) | 53°23.593′ | 107°24.837′ |
| 21 | 3 km from the Ukhan Cape | 53°02.973′ | 107°25.348′ |
| 22 | central station of section the Ukhan Cape−Tonkii Cape | 52°53.726′ | 107°31.915′ |
| 23 | 3 km from the Tonkii Cape | 52°44.582′ | 107°38.475′ |
| 24 | Shunte Pravyi Cape | 53°17.148′ | 107°48.276′ |
| 25 | Barguzinskii Bay | 53°27.230′ | 108 44.294′ |
| 26 | Chivyrkuiskii Bay | 53°49.652′ | 109°08.163′ |
| 27 | central station of section the Pokoiniki Cape–Great Ushkanii Island | 53°56.514′ | 108°25.905′ |
| 28 | central station of section the Cape Zavorotnyi−Sosnovka River | 54°14.595′ | 108 59.828′ |
| 29 | 3 km from Davsha village | 54°22.657′ | 109°25.217′ |
| 30 | central station of section the Cape Elokhin−Davsha village | 54°27.143′ | 109 03.943′ |
| 31 | central station of section the Kotelnikovskii Cape−Amnundakan River | 55°02.738′ | 109°26.028′ |
| 32 | 3 km from the Turali Cape | 55°17.643′ | 109°42.805′ |
| 33 | central station of section Baikalskoe village−Turali Cape | 55°19.228′ | 109°28.933′ |
| 34 | 3 km from Baikalskoe village | 55°21.005′ | 109°14.853′ |
| 35 | Ayaya Bay | 55°27.211′ | 109°46.836′ |
| 36 | Frolikha Bay | 55°32.937′ | 109°47.504′ |
| 37 | opposite Frolikha Bay | 55°32.073′ | 109°43.929′ |
| 38 | central station of section Tyya River−Nemnyanka Cape | 55°34.064′ | 109°35.660′ |
| 39 | Birakan Cape | 55°37.444′ | 109°50.766′ |
| 40 | 5 km from the Verkhnyaya Angara River | 55°40.665′ | 109°49.607′ |
| 41 | Angara-Kicher shoal (Millionnyi island) | 55°43.607′ | 109°44.702′ |
| 42 | 7 km from Nizhneangarsk city | 55°43.174′ | 109°37.842′ |
